# The Safety of Endoscopic Procedures in Patients With Thrombocytopenia: A Systematic Review and Meta-Analysis

**DOI:** 10.7759/cureus.51773

**Published:** 2024-01-06

**Authors:** Akash Patel, Guy Treves, Isha Samreen

**Affiliations:** 1 Internal Medicine, Eisenhower Health, Rancho Mirage, USA; 2 Internal Medicine, Hemet Global Medical Center, Hemet, USA

**Keywords:** post-endoscopy complications, gastrointestinal endoscopy, systematic review and meta analysis, endoscopic procedures, thrombocytopenia

## Abstract

Endoscopic procedures are essential in gastroenterology but pose significant risks for thrombocytopenic patients who have lower platelet counts, increasing the likelihood of bleeding complications. This systematic review and meta-analysis followed Preferred Reporting Items for Systematic Reviews and Meta-Analyses (PRISMA) guidelines to assess bleeding risks in thrombocytopenic patients undergoing various endoscopic procedures. A comprehensive search was conducted across databases like PubMed, MEDLINE, and EBSCO, using stringent criteria for inclusion and exclusion, with study quality assessed via the Newcastle-Ottawa Scale and thrombocytopenia severity classified by Common Terminology Criteria for Adverse Events (CTCAE) criteria. Statistical analysis focused on bleeding event incidence rates at different platelet count thresholds, utilizing RevMan Web (Cochrane, London, UK) and Excel (Microsoft® Corp., Redmond, WA). The search yielded 1,675 potential articles, but only three retrospective cohort studies were selected. Results showed a significant increase in bleeding risk for patients with platelet counts below 50,000/mm^3^, particularly under 25,000/mm^3^, with a 5.5% prevalence of post-procedure bleeding in moderate to severe thrombocytopenic patients versus 4.0% in those with higher counts, and a threefold higher risk in severe thrombocytopenia. The study highlights the need for meticulous pre-procedure assessments in thrombocytopenic patients and points out disparities in guideline recommendations, suggesting personalized approaches based on patient-specific risks. It underscores balancing diagnostic yield against bleeding risks, especially in severe thrombocytopenia, and discusses the controversial role of prophylactic platelet transfusions, advocating for a nuanced approach. In conclusion, this meta-analysis provides critical insights into managing thrombocytopenia in endoscopic procedures, emphasizing the importance of individualized patient assessment and adherence to evolving guidelines, and underlining the necessity of further research to refine these guidelines and improve patient safety and outcomes in this challenging clinical scenario.

## Introduction and background

Endoscopic procedures are increasingly employed in diagnosing and managing various gastrointestinal (GI) disorders. Despite their widespread utility, these procedures can pose significant risks, particularly in patients with hematologic abnormalities like thrombocytopenia which is characterized by an abnormally low platelet count, and increases the risk of bleeding, a complication that can be exacerbated during endoscopic interventions [1,2]. The condition can arise from a multitude of causes, including chronic illnesses, medication effects, and bone marrow disorders, each contributing differently to the risk profile of endoscopic procedures [2]. The prevalence of thrombocytopenia in patients undergoing endoscopic procedures varies, and its impact on procedural safety remains a critical area of investigation [3]. The management of these patients poses a significant challenge, balancing the need for accurate diagnosis and therapeutic intervention against the potential for serious bleeding complications.

Previous studies have highlighted the increased risk of bleeding in thrombocytopenic patients undergoing endoscopic procedures. However, these studies have often been limited by small sample sizes, lack of standardization in defining thrombocytopenia severity, and variability in endoscopic practices [4]. Furthermore, there is a paucity of literature providing a stratified analysis of bleeding risks based on different platelet count thresholds, which is crucial for clinical decision-making.

The Common Terminology Criteria for Adverse Events (CTCAE) provides a standardized framework for classifying adverse events, including thrombocytopenia, in clinical trials [5]. This classification system offers an opportunity to uniformly define thrombocytopenia severity across different studies, allowing for a more comprehensive and comparative analysis of associated bleeding risks [5].

The present systematic review and meta-analysis aim to fill these gaps by quantifying the risk of bleeding associated with endoscopic procedures in thrombocytopenic patients. By applying rigorous selection criteria and quality assessment aligned with the Preferred Reporting Items for Systematic Reviews and Meta-Analyses (PRISMA) guidelines, this study synthesizes available evidence to provide a clearer understanding of the bleeding risks stratified by thrombocytopenia severity. Additionally, it explores the impact of various endoscopic procedures on these risks, offering vital insights into the safe management of this vulnerable patient population.

In doing so, this research not only contributes to the existing body of knowledge but also offers an evidence-based guide for risk stratification and decision-making processes. Understanding the nuances of bleeding risks associated with different levels of thrombocytopenia can significantly enhance patient safety and inform the development of tailored management strategies for such patients who require endoscopic evaluation.

## Review

Methods

Search Strategy and Selection Criteria

The present systematic review was conducted in alignment with the PRISMA guidelines. A thorough search was conducted using several databases, including PubMed, MEDLINE, and EBSCO, to identify relevant studies. The search terms were strategically chosen to encompass both thrombocytopenia ("Low platelets" OR "Thrombocytopenia") and various endoscopic procedures ("Endoscopy" OR "Esophagogastroduodenoscopy" OR "Colonoscopy" OR "ERCP" OR "Gastrointestinal endoscopy" OR "Endosc*"). The focus of the studies was specifically on studies that addressed the intersection of these terms. We excluded review articles, systematic reviews, meta-analyses, case reports, conference abstracts, preprint articles, non-English texts, and articles without full texts. The software HubMeta (HubMeta, Calgary, Canada) was utilized for title and abstract screening.

Quality Assessment

Quality assessment of the selected studies was rigorously conducted using the Newcastle-Ottawa Scale (NOS). This process was independently carried out by two reviewers, with discrepancies resolved through mutual discussion, ensuring a fair and unbiased evaluation of the study quality.

Data Extraction

The following key information was gathered from each study: participant characteristics, study location, authorship, and publication year. A focal point was patient thrombocytopenia characteristics. The severity of thrombocytopenia was categorized according to the CTCAE classification. Specifically, severe thrombocytopenia (grade 4) was defined as platelet counts less than 25,000/mm^3^, moderate thrombocytopenia (grade 3) as counts between 25,000 and 50,000/mm^3^, and mild thrombocytopenia (grade 2) as counts ranging from 50,000 to 75,000/mm^3^. This method ensured consistency with the present comparative analysis. Alongside this, we collected data on endoscopic procedures, demographics, and outcomes like bleeding events and mortality rates. This comprehensive approach allowed for a detailed evaluation of the safety and outcomes of endoscopic procedures in patients with varying degrees of thrombocytopenia.

Statistical Analysis

The statistical analysis was conducted using RevMan Web (Cochrane, London, UK) and Excel (Microsoft® Corp., Redmond, WA). The random-effects model was employed to accommodate the expected heterogeneity among studies. Heterogeneity was assessed using the I² statistic and the Chi-square test. The risk of bias in individual studies was evaluated, and sensitivity analyses were conducted to test the robustness of our findings. Meta-regression analyses were planned to explore potential sources of heterogeneity, such as patient age, severity of thrombocytopenia, and types of endoscopic procedures. We also assessed publication bias using funnel plots and Egger's regression test. Data synthesis involved calculating summary estimates of incidence rates with 95% confidence intervals and was presented in forest plots.

Results

Search Results

**Figure 1:**
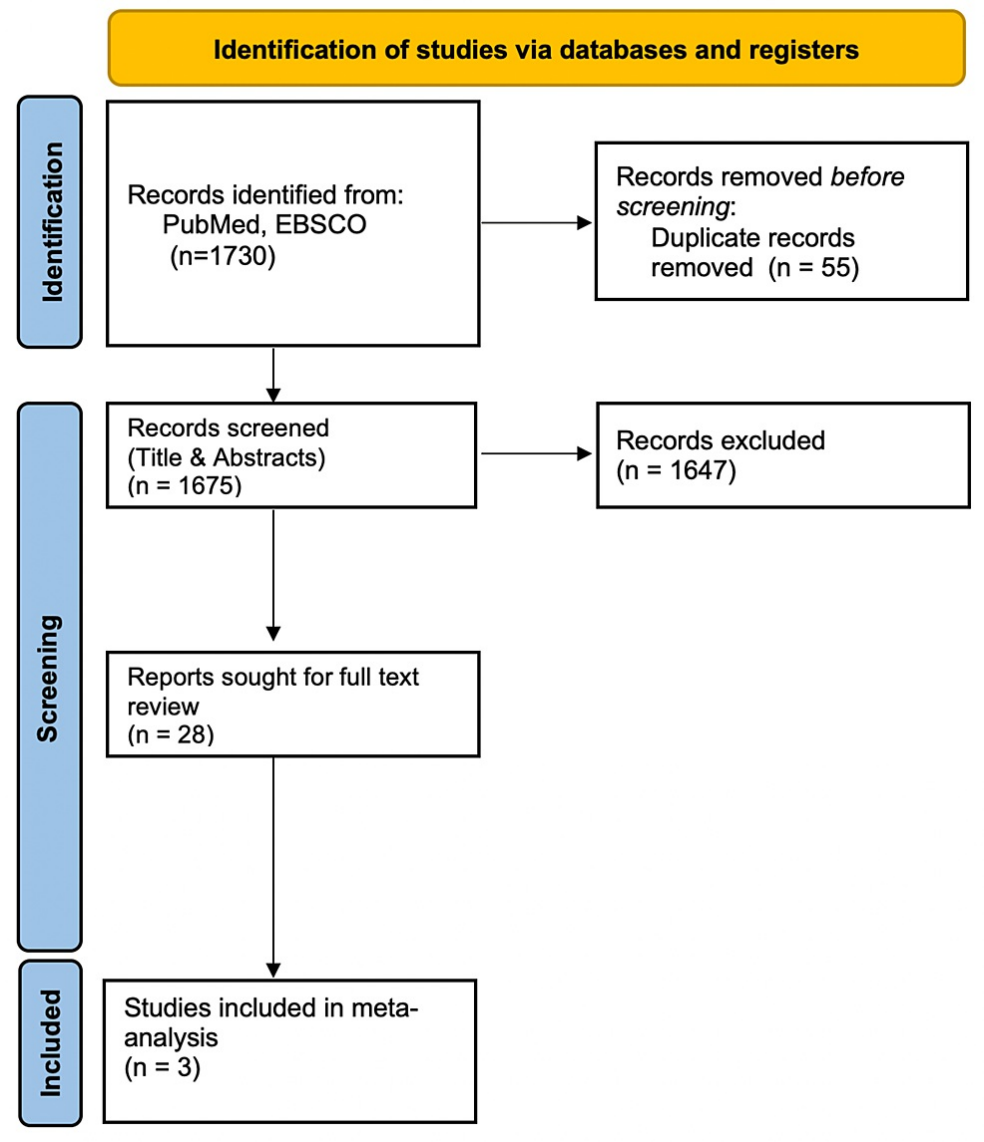
PRISMA Chart PRISMA: Preferred Reporting Items for Systematic Reviews and Meta-Analyses

In the study search strategy across multiple databases aligned with the PRISMA guidelines. The search was structured around two primary terms: thrombocytopenia, represented as "Low platelets" OR "Thrombocytopenia", yielding 143,679 records, and endoscopic procedures, represented as "Endoscopy" OR "Esophagogastroduodenoscopy" OR "Colonoscopy" OR "ERCP" OR "Gastrointestinal endoscopy" OR "Endosc*", resulting in 573,510 records. The combined search identified 1,675 articles for potential inclusion (Figure 1).

The initial title and abstract screening of these articles led to the exclusion of 715 manuscripts, primarily due to their nature as review articles, systematic reviews, meta-analyses, or case reports, leaving 960 articles. Further selection based on full-text screening, while considering exclusion criteria, ultimately resulted in the inclusion of three articles in our final analysis.

Study and Participant Characteristics

In our review, three retrospective cohort studies were selected, characterized by diverse patient demographics and thrombocytopenia severity categories. The studies, detailed in Table 1, encompass a broad spectrum of patient ages, genders, and ethnic backgrounds, with comprehensive data on the outcomes of endoscopic procedures in thrombocytopenic patients.

**Table 1:** Study Characteristics

Author and Year	Study Type	Inclusion Criteria: Diagnosis/Condition	Total Population	Mean Age	Gender Distribution: Male %	Bleeding Events: Grade 4	Bleeding Events: Grade 3	Bleeding Events: Grade 2	Bleeding Events: Grade 1
Abu-Sbeih et al., 2018 [6]	Retrospective cohort study	Diagnosis of malignancy	588 patients	58 years	58.5%	21/200	11/260	4/148	N/A
Oh et al., 2016 [7]	Retrospective case control study	Case: Patients with chronic hematologic thrombocytopenia (Aplastic anemia/ITP). Control: Age-, sex-, procedure-matched patients.	458 patients	52 years	~46%	2/9	7/24	2/39	6/103
Krishna et al., 2014 [4]	Retrospective cohort study	Thrombocytopenia with platelet <75K	395 patients	54.8 years	56.9%	1/60	5/299	2/56	N/A

Quality Assessment

The quality of the included studies was rigorously assessed using the NOS and summarized in Table 2.

**Table 2:** Newcastle-Ottawa Scale for Included Studies

Criteria	Abu-Sbeih et al., 2018 [6]	Oh et al., 2016 [7]	Krishna et al., 2014 [4]
Representativeness of Exposed Cohort	1	1	1
Selection of Non-exposed Cohort	N/A	1	N/A
Ascertainment of Exposure	1	1	1
Outcome Not Present at Start	1	1	1
Comparability	2	2	1
Assessment of Outcome	1	1	1
Follow-Up Long Enough for Outcomes	1	1	1
Adequacy of Follow-Up of Cohorts	1	1	1
Total Score	8	9	7
Overall Quality	High	High	Moderate to High

Prevalence of Post-procedure Bleeding in Moderate to Severe Thrombocytopenia

Our meta-analysis quantified the prevalence of post-procedure bleeding in cases of moderate to severe thrombocytopenia. Within the patient cohort of moderate to severe thrombocytopenia (n=852), 47 individuals manifested post-procedure bleeding, yielding a prevalence of 5.5%. Also, among a comparison group of 346 patients with platelet counts greater than 50,000/mm^3^, there was a 4.0% prevalence of bleeding (14 patients).

Comparative Risk of Bleeding Based on Platelet Thresholds

**Figure 2:**
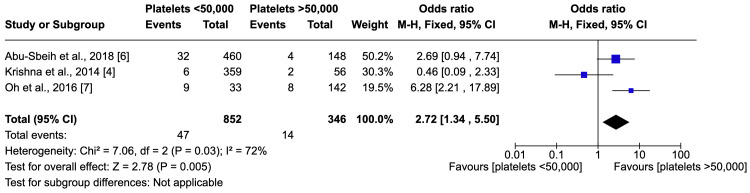
Comparative Risk of Bleeding Based on Platelet Thresholds

Bleeding outcomes were also compared between patients with platelet counts in grades 3 and 4, and those above this demarcation, as shown in Figure 2. The range of odds ratios for bleeding events spanned from 0.46 to 6.28 across the studies. Meta-analysis revealed significantly higher odds of bleeding in grades 3 and 4, with an odds ratio of 2.72 (95% CI: 1.34-5.50). Despite the moderate heterogeneity across studies (I² = 72%), the analysis indicates a substantial increase in bleeding risk among patients with lower platelet counts.

Assessment of Bleeding in Severe vs. Mild Thrombocytopenia

**Figure 3:**
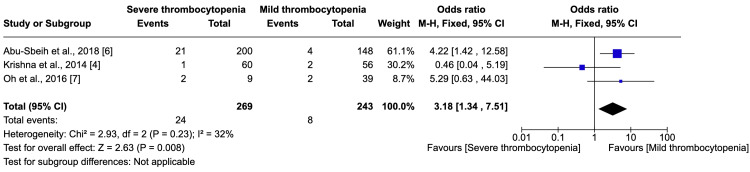
Assessment of Bleeding in Severe vs. Mild Thrombocytopenia

The incidence of bleeding events was 8.9% in severe thrombocytopenia cases compared to 3.3% in mild cases, a statistically significant discrepancy (p=0.008). An odds ratio of 3.18 (95% CI: 1.34-7.51) suggested a tripling of bleeding risk in severe thrombocytopenia. This analysis, incorporating moderate heterogeneity (I² = 32%), is graphically represented in Figure 3.

Discussion

Thrombocytopenia presents a unique challenge in endoscopic procedures, balancing the necessity of the procedure against the increased risk of bleeding. The current meta-analysis sheds light on this intricate balance, emphasizing the need for meticulous pre-procedure assessment and vigilant management as bleeding risk is significantly elevated in patients with moderate and severe thrombocytopenia.

The importance of assessing platelet counts and coagulation status before endoscopic procedures cannot be overstated. Current American Society of Oncology guidelines recommend a platelet count threshold of greater than 50,000/mL in the absence of coagulation abnormalities for invasive procedures [8]. Our findings, which suggest heightened bleeding risks in patients with platelet counts below this threshold, are consistent with these guidelines. The American Society for Gastrointestinal Endoscopy (ASGE), however, indicates that there is no established minimum threshold for diagnostic upper GI endoscopy, with some authors suggesting 20,000/mL as a safe lower limit [9-11]. The disparity in these recommendations highlights the need for a personalized approach, considering individual patient risks and the nature of the procedure.

Of particular note, patients who suffer from pathologies that are associated with thrombocytopenia should be carefully considered for bleeding risk when evaluated for endoscopic procedures as the current study demonstrates a greatly increased incidence of bleeding in grade 4 thrombocytopenia. This finding is particularly relevant for patients with underlying conditions like hematological malignancies or advanced liver disease, where thrombocytopenia is often a concomitant challenge [12]. In light of this, endoscopists must be prepared for higher bleeding risks in these patient populations.

The management of post-biopsy bleeding in thrombocytopenic patients is a critical aspect of endoscopic care. The analysis of the present study suggests that while the risk is higher, it is not prohibitive, necessitating careful monitoring and preparedness for intervention. The decision to proceed with a biopsy in thrombocytopenic patients should be guided by the potential diagnostic yield versus the risk of bleeding and evaluated on a case-by-case basis. Among the included studies, Krishna et al. [4] and Oh et al. [7] assessed for post-biopsy bleeding with the incidence of 1.16% and 26% bleeding, respectively for patients with platelets <50,000.

The use of prophylactic platelet transfusions remains controversial. While some guidelines suggest their use in severe thrombocytopenia, our findings indicate a more nuanced approach may be required [13]. The risk of transfusion-related complications must be weighed against the potential benefit of preventing bleeding, especially in cases where platelet counts are marginally below the recommended thresholds.

There are several limitations to our study. The nature of included studies and potential variability in practice across different institutions may influence the generalizability of our findings. Future research, ideally in the form of randomized controlled trials, is needed to establish more definitive platelet count thresholds and evaluate the role of prophylactic treatments in thrombocytopenic patients undergoing endoscopic procedures.

## Conclusions

The management of thrombocytopenia in patients undergoing endoscopic procedures requires a balance of risk and benefit, guided by a thorough understanding of the patient's condition and careful consideration of current guidelines. As the field advances, ongoing research and refinement of guidelines will be crucial in optimizing patient outcomes and safety in this challenging clinical scenario. At this time there does not seem to be a guideline consensus and therefore the decision to proceed with endoscopy in a thrombocytopenic patient will require a comprehensive individualized evaluation.

## References

[1] Elsaid MI, Rustgi VK, Loo N, Aggarwal K, Li-McLeod J, Niu X, Poordad F (2020). The burden associated with thrombocytopenia and platelet transfusions among patients with chronic liver disease. J Med Econ.

[2] Peck-Radosavljevic M (2000). Thrombocytopenia in liver disease. Can J Gastroenterol.

[3] Giannini EG, Greco A, Marenco S, Andorno E, Valente U, Savarino V (2010). Incidence of bleeding following invasive procedures in patients with thrombocytopenia and advanced liver disease. Clin Gastroenterol Hepatol.

[4] Krishna SG, Rao BB, Thirumurthi S, Lee JH, Ramireddy S, Guindani M, Ross WA (2014). Safety of endoscopic interventions in patients with thrombocytopenia. Gastrointest Endosc.

[5] Freites-Martinez A, Santana N, Arias-Santiago S, Viera A (2021). Using the Common Terminology Criteria for Adverse Events (CTCAE - version 5.0) to evaluate the severity of adverse events of anticancer therapies. Actas Dermosifiliogr (Engl Ed).

[6] Abu-Sbeih H, Ali FS, Coronel E (2019). Safety of endoscopy in cancer patients with thrombocytopenia and neutropenia. Gastrointest Endosc.

[7] Oh HJ, Park JM, Yoon SB (2017). Bleeding after endoscopic procedures in patients with chronic hematologic thrombocytopenia. Dig Dis Sci.

[8] Schiffer CA, Anderson KC, Bennett CL (2001). Platelet transfusion for patients with cancer: clinical practice guidelines of the American Society of Clinical Oncology. J Clin Oncol.

[9] Ben-Menachem T, Decker GA, Early DS (2012). Adverse events of upper GI endoscopy. Gastrointest Endosc.

[10] Nilles KM, Caldwell SH, Flamm SL (2019). Thrombocytopenia and procedural prophylaxis in the era of thrombopoietin receptor agonists. Hepatol Commun.

[11] Biolato M, Vitale F, Galasso T, Gasbarrini A, Grieco A (2023). Minimum platelet count threshold before invasive procedures in cirrhosis: evolution of the guidelines. World J Gastrointest Surg.

[12] Desborough M, Estcourt LJ, Doree C, Trivella M, Hopewell S, Stanworth SJ, Murphy MF (2016). Alternatives, and adjuncts, to prophylactic platelet transfusion for people with haematological malignancies undergoing intensive chemotherapy or stem cell transplantation. Cochrane Database Syst Rev.

[13] Nomoto H, Morimoto N, Miura K (2020). Lusutrombopag is effective and safe in patients with chronic liver disease and severe thrombocytopenia: a multicenter retrospective study. BMC Gastroenterol.

